# Analysis of lung cancer measures of the National Cancer Network pilot study in Poland for potential improvement in the quality of advanced-stage lung cancer therapy

**DOI:** 10.1186/s12885-021-08994-z

**Published:** 2021-11-20

**Authors:** Łukasz Trembecki, Aleksandra Sztuder, Ireneusz Pawlak, Rafał Matkowski, Adam Maciejczyk

**Affiliations:** 1 Department of Oncology, Wroclaw Medical University, pl. L. Hirszfelda 12, 53-413 Wroclaw, Poland; 2 Department of Radiotherapy, Wroclaw Comprehensive Cancer Center, pl. L. Hirszfelda 12, 53- 413 Wroclaw, Poland; 3 Department of Thoracic Surgery, Wroclaw Comprehensive Cancer Center, pl. L. Hirszfelda 12, 53-413 Wroclaw, Poland; 4 Wroclaw Comprehensive Cancer Center, Breast Unit, pl. L. Hirszfelda 12, 53-413 Wroclaw, Poland

**Keywords:** Lung cancer, Quality measures, Targeted therapy, Poland

## Abstract

**Background:**

This study aimed to present the performance of the National Cancer Network’s (NCN) pilot program in the Lower Silesian Voivodeship (southwestern province of Poland with a population of 2,9 million in 2019), to analyse measures describing lung cancer patients and to determine whether those measures can be used to improve the treatment outcomes of stage III and IV patients with lung cancer in Poland.

**Methods:**

Three measures of the NCN pilot programme were analysed: “Percentage of patients with genetic and molecular testing for predictive factors”, “Assessment of the completeness of a pathological examination”, and “Percentage of stage III and IV cancer patients”. As many as 2,218 patients with ICD-10-CM Diagnosis Code C34 were included in the NCN pilot program from 1 to 2019 to 31 December 2020, in the Lower Silesian Voivodeship. The scores of each measure were calculated quarterly by the Regional Coordinating Centre, Wroclaw Comprehensive Cancer Center, Wroclaw, Poland.

**Results:**

Genetic and molecular testing among stage III and IV patients was performed in only 40% and 60% of patients, respectively. The incompleteness of histopathological examinations did not exceed 0.5%. Stage III and stage IV patients accounted for 37% and 35% of the analysed patients, respectively.

**Conclusions:**

The NCN pilot program measures presented in this study appear to be highly sensitive, simple, and transparent tools to monitor the quality of lung cancer diagnosis and assess clinical staging in patients within a specific region.

An increase in the proportion of stage III and IV patients who will undergo genetic and molecular testing in the era of modern drug therapies should result in improved treatment outcomes in this patient group. In the present analysis, the values of the main analysed measure, which evaluates the number of genetic and molecular tests for predictive factors for lung cancer, were subject to significant fluctuations during the pilot project. Both upwards and downwards trends were observed. Further analysis in the future is warranted to eliminate the unfavourable factors influencing the obtained values of the measure.

**Supplementary Information:**

The online version contains supplementary material available at 10.1186/s12885-021-08994-z.

## Introduction

Cancer is currently the largest organisational and economic challenge facing the health care system in Poland in the third decade of the 21st century.

Demographic data show a progressive change in the Polish population structure, with an increasing proportion of persons aged older than 60 years. The proportion of the elderly population is predicted to reach 40% by 2050 [1].

The abovementioned trend coincides with an observed increase in the incidence of malignant neoplasms. By 2040, according to WHO data, the incidence of malignant neoplasms in Poland is expected to increase by 20%. In the over-60 age group, it may even exceed 30% [2].

Currently, lung cancer is the most frequently diagnosed malignant neoplasm in Poland. It accounts for more than 15% of all oncologic diagnoses and causes nearly one-quarter of all deaths from malignant neoplasms. A steady increase in new cases is predicted over the next decades (by 2040) [2]. Given the abovementioned data, lung cancer was included in a pilot project of the Polish National Oncology Network (Krajowa Sieć Onkologiczna – KSO). Under the 2018 Regulation of the Ministry of Health, Holy Cross Voivodeships (southeastern province of Poland with a population of 1,2 million in 2019) and Lower Silesian Voivodeships (southwestern province of Poland with a population of 2,9 million in 2019) were included in the project. The pilot study covered lung, breast, colorectal, prostate and ovarian malignancies. The main objective of the project was to analyse the activities enabling comprehensive and coordinated cancer care in selected voivodeships.

The study aimed to present the results of the KSO pilot study in the Lower Silesian Voivodeship in terms of completeness and quality of pathomorphological and molecular diagnostics of lung cancer. Particular emphasis was placed on molecular and genetic predictive factors for that type of cancer—activating mutations in the EGFR gene, ALK and/or ROS1 gene rearrangement and evaluation of PD-L1 expression. These criteria were considered essential for lung cancer and were selected after analysing the causes of suboptimal lung cancer treatment results and correspond to recent lung cancer treatment guidelines [3–5].

The improvement in these indicators, under the supervision of the KSOs, should automatically contribute to improving the quality of treatment and positively influencing treatment results.

## Materials and methods

The KSO pilot study was presented at the end of 2018 under the Regulation of the Ministry of Health on the pilot program for patient care within the cancer network [6].

The network should be considered an organisational structure in the voivodeship, which includes the Regional Coordinating Centre (RCC) and Level I and II centres included in the pilot study. The criteria for inclusion of a centre in the pilot study as part of the network are based on quality and organisational factors, including the minimal number of cancer treatment procedures, chemotherapy and radiotherapy services performed annually. The highest values in providing specific cancer treatment services constitute a major criterion that determines the RCC allocation. [7]

The Wroclaw Comprehensive Cancer Center in Wroclaw fulfilled the requirements for obtaining the RCC position in the voivodeship. Fifteen facilities were recognised as Level I and II Centres.

The role and services of each unit in the pilot study were defined in accordance with the aforementioned regulation. In 2019, two additional voivodeships, Podlaskie and Pomeranian Voivodeships, were included in the pilot study [8]. The completion date of the project was set at 31 December 2021 [9].

One of the main objectives of the KSO pilot study was to establish a monitoring system of the quality of care for cancer patients. Other goals of this program were, among others, improving treatment outcomes, reducing inequalities in access to cancer care, reducing the number of complications, or increasing funding for highly specialised centres. For this purpose, 35 measures were designed. The Additional file 1: Table S1 provides a description of all indicators used in Polish National Oncology Network. Three measures can be distinguished: internal process measures, statistical measures, and measures describing the patient health status. The measures were suggested by the Ministry of Health based on public consultations and after considering the opinions and comments made by communities involved in cancer care. To provide a uniform model for the calculation of measures across centres, evaluation forms were developed. They include a definition of the process to which they relate, a measure’s structure, instructions to determine measures, data sources and target value (PROQUAL Management Institute).

The Regional Coordinating Centres (RCCs), which collect clinical and billing data received both from the centres participating in the pilot study and the National Health Fund (NHF), are involved in qualitative analysis—i.e., the calculation of measures. The entire data collection process is based on a central data warehouse, where information is collected in a uniform IT format. This format enables the use of analytics software (MedStream Designer; Transition Technologies) to group and select specific data. These data are then used to calculate measures based on the evaluation forms.

Graphical presentation of the calculated measures is performed using a web application (Oncoindi v3.0).

The following measures were analysed in the present study: “Percentage of patients with genetic and molecular testing for predictive factors” (F_11_2 Measure Form) “Assessment of the completeness of a pathological examination” (F_10 Measure Form) and “Percentage of stage III and IV cancer patients” (F_9 Measure Form). Cytology alone or the lack of immunohistochemical examination was considered incomplete histopathological examination.

The obtained data concerned patients diagnosed with lung cancer. The calculation formula for the F_11_2 measure is the ratio of the number of all patients with ICD-10-CM Diagnosis Code C34 who were included in the pilot study, were diagnosed with stage III and IV non-small cell lung cancer, had a WHO general status of 0–2, and had undergone genetic and molecular testing, to the number of all patients with ICD-10-CM Diagnosis Code C34 who were included in the pilot study, were diagnosed with stage III and IV non-small cell lung cancer, and had a WHO general status of 0–2. Genetic and molecular testing concerned activating mutations in the EGFR gene and/or ALK and/or ROS1 gene rearrangement and/or immunohistochemical evaluation of PD-L1 expression. The expected values according to the measurement form were set at 100% for stage III and IV cancer. The calculation formulas for other measures are shown in Table 1. The data necessary to calculate the measures originated from the NHF’s xml reports and patient’s Hospital Information System (HIS) and covered the period from 1 to 2019 to 31 December 2020.

**Table 1:** Calculation formulas for F_9 and F_10 measures

Indicator	Percentage of stage III and IV cancer patients (F_9)	Assessment of the completeness of a pathological examination (F_10)
**Calculation formula** **(**quotient)	**Nominator** the number of patients included in the pilot with stage III and IV of a given cancer **Denominator**: the number of all patients included in the pilot with a given diagnosis and stage	**Nominator**: the number of all completed oncological diagnostics assessment cards in individual neoplasms included in the pilot study in which incompleteness was found **Denominator**: the number of all completed oncological diagnostics assessment cards (regardless of the assessment performed) in each of the pilot cancers.
**Expected value of the indicator**:	0%	0%
**Type of the indicator**	statistics (independent of the organization)	Internal processes indicator (quality)

The results are published quarterly, and the graphical presentation is performed on a cumulative basis against previous quarters.

## Results

A total of 2,218 patients with ICD-10-CM Diagnosis Code C34 were included in the KSO pilot study in the Lower Silesian Voivodeship from 1 to 2019 to 31 December 2020.

The age structure of patients showed that nearly 70% of patients were aged 60-74 years. Most of the patients were aged 65-69 years (27%; n=609).

The F_11_2 measure, which evaluates the number of genetic and molecular tests for predictive factors for lung cancer, was analysed in detail. The data originated from 5 centres of the Lower Silesian Voivodeship. The results of the measure in question have been reported since the second quarter of 2019.

**Fig. 1:**
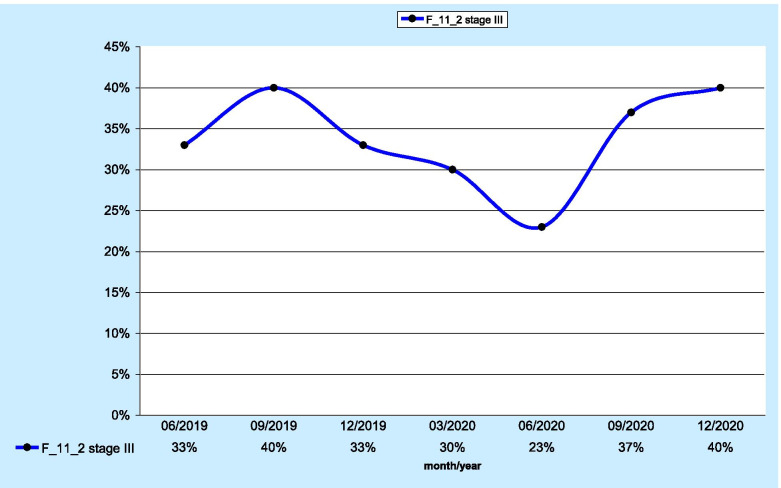
Percentage of stage III lung cancer patients with genetic and molecular testing

The F_11_2 measure was applied to evaluate 165 stage III patients. Genetic and molecular testing was performed in 40% of patients (n=67; Fig. 1). Aggregate data were collected from all the centres. The highest percentage of tests was recorded at the Lower Silesian Centre of Lung Diseases in Wroclaw (44%; n=47).

**Fig. 2:**
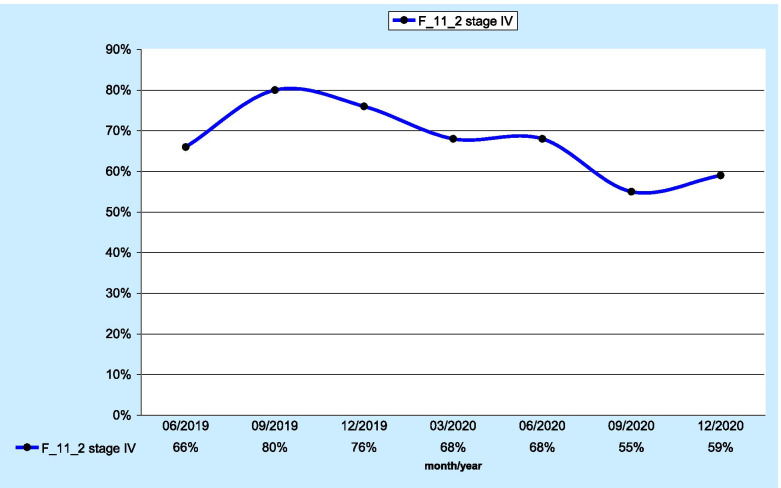
Percentage of stage IV lung cancer patients with genetic and molecular testing

The pilot study included 142 stage IV patients who were evaluated by the aforementioned measure. In that patient group, the F_11_2 score was 60% (n=84; Fig. 2). The maximum score was reported at the Multispecialty Hospital in Zgorzelec. The score was 100%; however, the analysis involved only one pilot patient. The second-highest score in voivodeship—64%—was reported by the Wroclaw Comprehensive Cancer Center in Wroclaw and the Silesian Centre of Lung Diseases in Wroclaw. The number of patients who had undergone molecular testing was 25 and 48, respectively.

When assessing the results for individual quarters, significant fluctuations in the values of the F_12_2 measure were shown. In the case of patients in stage III, after an initial decrease, a significant increase was found from the third quarter of 2020, up to the maximum value reached at the end of 2020. For the group of patients in stage IV, a downwards trend was observed since the end of 2019.

Subsequently, the completeness of a pathological examination (F_10 measure) was measured.

As part of the initial diagnostic procedure, 1,247 completed oncological diagnostic evaluation forms with a lung cancer diagnosis were analysed. In one case, the result of the pathological examination was incomplete, representing 0.08% of all cases. For the evaluation of the F_10 measure at the stage of in-depth diagnosis, 1,120 evaluation forms were evaluated. The measure score was 0.5% (n=5).

**Fig. 3:**
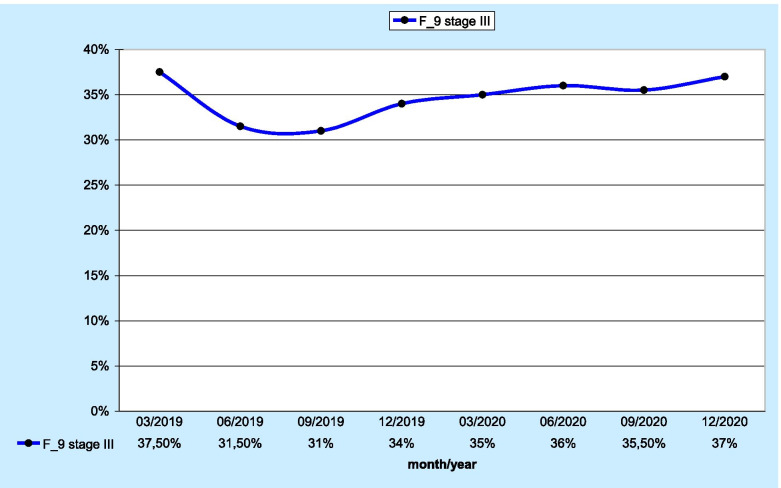
Percentage of stage III lung cancer patients

**Fig. 4:**
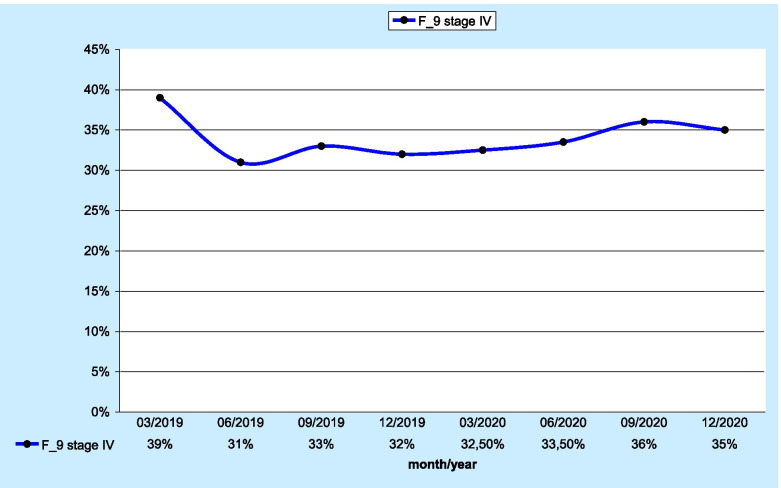
Percentage of stage IV lung cancer patients

The last analysed parameter was the F_9 measure describing the percentage of patients diagnosed with stage III and IV lung cancer. As many as 1,921 patients were included in the evaluation. Stage III and IV patients accounted for 37% (n=709) and 35% (n=678) of all patients, respectively. Those data are shown in Figs. 3 and 4, respectively.

## Discussion

The present study describes the design and basis of the KSO pilot study in the Lower Silesian Voivodeship and focuses on three measures related to patients diagnosed with lung cancer.

The analysis of the age structure of the pilot patients shows a definite predominance of patients in the 60-74 age range. This finding confirms the latest epidemiological data and agrees with the observed trend of the increasing incidence of malignant neoplasms in the abovementioned age group [10].

A significant score was reported for a measure concerning the percentage of stage III and IV lung cancer patients, indicating that 72% of patients included in that evaluation were diagnosed at a significantly advanced stage of the disease.

The information obtained through the aforementioned tool provides accurate insight into the current situation in terms of disease advancement in patients within a specific area. The measure in question appears to be a very sensitive indicator that may also be used to evaluate health care interventions aimed at decreasing the number of stage III and IV patients. A change in the current proportion in Poland would result in an improvement in lung cancer treatment outcomes, which remain unsatisfactory. The main reason for the low cure rate is the predominance of advanced-stage patients seeking cancer treatment. For example, in the Lower Silesian Voivodeship, the overall 3-year relative survival rate of lung cancer patients does not exceed 20% [11].

In the present study, the main objective was to evaluate the measure that defines the percentage of molecular and genetic testing in lung cancer diagnosis and attempt to demonstrate its usefulness in improving the treatment efficacy in patients with advanced cancer.

Concerning the percentage of molecular tests performed, 40% in stage III and 60% in stage IV are comparable to other similar reports. Vrijens et al. reported EGFR testing at the 53% level using a quality indicator [12].

The results obtained from the KSO pilot analysis regarding the discussed measure indicate that, among stage III patients, an increase was observed in the procedures performed since 06/2020; among stage IV patients, an unfavourable downwards trend was noted. From 09/2019 to 12/2020, a decrease of nearly 20% was found in the performed molecular and genetic tests.

One of the hypotheses explaining this result may be the influence of the SARS-CoV-2 epidemic on the functioning of oncological centres, including the departments of pathomorphology and molecular diagnosis. Further analysis is needed to identify the causes of suboptimal outcomes.

According to our assessment results of the pathological examination completion measure, the measure can also be used as a highly sensitive indicator, markedly supplementing the control of the discussed stage of diagnosis.

Importantly, correct and accurate data reporting (TNM classification) intended to calculate the measure in question is critical in this case. Evaluating the pilot data presented in the present study showed a difference between the number of patients included in the pilot study and number included in the evaluation of the measure in terms of clinical staging (n=1921). The reason is likely that, for some patients, the date of pilot study enrolment does not coincide with the date on which the clinical stage is determined. Therefore, the clinical stage was reported in that patient group after the period covering the aforementioned analysis. The significant impact of human error and errors in reporting of the data concerned cannot be excluded either.

Indirectly, information of that type may be used to monitor the quality and completeness of the information collected at the Coordinating Centres and enable a quick response to improve the process.

Significant progress in terms of systemic treatment, which results in improved treatment outcomes of advanced-stage patients, and simultaneous availability of those therapies for Polish patients under drug programs, is vital considering that stage III and IV patients are the largest group of lung cancer patients in Poland [5]. The importance of the departments of pathomorphology and molecular diagnosis of cancer cannot be ignored in the diagnostic and therapeutic processes. The optimal use of potential next-generation drugs depends on the quality, equipment and experience of those units.

Hence, an increase in the number of molecular and genetic tests, both in a given centre and in a given region, is likely to result in improved therapeutic outcomes. This claim is directly related to the prognostic function of this testing for specific drugs.

At the same time, the measure enables monitoring of the current status of the abovementioned stage of diagnosis and provides a basis to implement organisational mechanisms to improve the quality of the provided service. Analyses from other countries describing developed quality measures for non-small cell lung cancer have led to convergent conclusions [13, 14].

Reliable comparisons between the centres are also possible through standardisation and transparency of the entire process of data collection and storage within KSO. Benchmarking is one of the elements that could be part of a health care system. Its concept would be based on rewarding the quality, comprehensiveness and coordination of services provided in oncology. In this system, payment for services would be strongly correlated with their quality, further contributing to strengthening the trends and mechanisms aimed at ongoing monitoring of the services. At the same time, KSO is consistent with the National Oncology Strategy 2020-2030, particularly regarding the improvement of the diagnostic procedure and cancer treatment, as well as the development and implementation of organisational changes that ensure coordinated and comprehensive cancer care [15].

The creation of a huge database with data collected in unified IT formats provides an opportunity to apply new data processing technologies, particularly *machine learning* and *data mining*, to identify new relationships between data [16].

The measures used in the KSO pilot study also have other advantages. Namely, their structure enables quick and relatively easy modification in terms of definition and calculation formula. Thus, the monitoring process can be adapted—for example, in terms of the molecular testing in question, when new, as yet unknown, predictive factors are diagnosed.

The NCN pilot program is a completely new approach implemented in the health care system in Poland. Therefore, resources and IT tools are currently unavailable so that this project can be used for comparative purposes. These factors are the main limitation of the presented study. Our results require comparisons with the data collected from the next period.

## Conclusions

The KSO pilot study measures presented in this study appear to be effective tools to monitor the quality of pathological diagnosis of lung cancer. Combined with predictive factors that are analysed through them, they can serve to improve treatment outcomes in patients with advanced cancer. This is possible due to an increase in the number of patients eligible for new drug therapies. Given emerging deficiencies in some areas of data reporting and measure calculation as part of KSO, the pilot study also provides an opportunity to identify such issues and address them.

Further studies on data comparison in future years are warranted.

## Supplementary Information

**Additional file 1.**

**Additional file 2.**

## Data Availability

The data underlying this article will be shared on reasonable request to the corresponding author.

## Abbreviations

NCN: National Cancer Network
KSO: Polish National Oncology Network (Krajowa Sieć Onkologiczna, polish)
EGFR: Epidermal growth factor receptor
ALK: Anaplastic lymphoma kinase
PD-L1: Programmed death-ligand 1
ROS1: c-ros oncogene 1
RCC: Regional Coordinating Centre
NHF: National Health Fund
IT: Information technology
HIS: Hospital Information System
