# Supplementary material for: Analysis of lung cancer measures of the National Cancer Network pilot study in Poland for potential improvement in the quality of advanced-stage lung cancer therapy
Source: BMC Cancer. 2021 Nov 20;21:1252. doi: 10.1186/s12885-021-08994-z (PMC8605770; doi:10.1186/s12885-021-08994-z)
Supplement: Supplementary file 1 — Additional file 1. [file 12885_2021_8994_MOESM1_ESM.doc]

| Indicator Description |
| --- |
| F_0 Multidisciplinary Tumor Boards assess the completeness of the diagnostics |
| F_1 The percentage of deaths within one year from the diagnosis of a malignant neoplasm, correlated to tumor stage |
| F_2 The percentage of deaths within 30 days from the date of surgery, correlated to tumor stage |
| F_3 Percentage of deaths within 30 days from the end of chemotherapy, correlated to tumor stage |
| F_4 Percentage of deaths within 30 days from the end of palliative radiotherapy, correlated to tumor stage |
| F_5 Percentage of patients requiring hospitalization due to complications after surgical treatment |
| F_6 Percentage of patients requiring re-hospitalization due to complications after radiotherapy |
| F_7 Percentage of patients requiring re-hospitalization due to complications after systemic treatment |
| F_8 Percentage of patients who received chemotherapy during inpatient hospitalization |
| **F_9* Percentage of stage III and IV cancer patients** |
| **F_10* Assessment of the completeness of a pathological examination** |
| F_11_1 Percentage of patients with genetic and molecular testing for predictive factors (colorectal cancer) |
| **F_11_2* Percentage of patients with genetic and molecular testing for**  **predictive factors (lung cancer)** |
| F_11_3 Percentage of patients with genetic and molecular testing for predictive factors - immunohistochemistry only (breast cancer) |
| F_11_4 Percentage of patients with genetic and molecular testing for predictive factors (breast cancer) |
| F_11_5 Percentage of patients with genetic and molecular testing for predictive factors - FISH only (breast cancer) |
| F_11_6 Percentage of patients with genetic and molecular testing for predictive factors (ovary cancer) |
| F_11_7 Percentage of patients with genetic and molecular testing for predictive factors - immunohistochemistry only (DCIS) |
| F_11_8 Percentage of patients with genetic and molecular testing for predictive factors (DCIS) |
| F_12_1 The percentage of surgical procedures performed with the minimally invasive surgery (colorectal cancer) |
| F_12_2 The percentage of surgical procedures performed with the minimally invasive surgery (lung cancer) |
| F_12_3 The percentage of surgical procedures performed with the minimally invasive surgery (ovary cancer) |
| F_12_4 The percentage of surgical procedures performed with the minimally invasive surgery (prostate cancer) |
| F_13 Median time elapsed from the date of registration of the patient for a diagnostic (imaging or pathomorphological) examination to the date of obtaining the result of this examination |
| F_14 Percentage of repeated diagnostic tests over a 6-week period  (computed tomography, endoscopy, biopsy, pathomorphological  assessment, molecular assessment), shown for each participating center  by tumor type and test type |
| F_15 Percentage of repeated surgical treatments in diagnosis other than  breast cancer |
| F_16 Percentage of patients with rectal cancer who received preoperative radiotherapy |
| F_17 Proportion of postoperative histopathology assessment in patients with colorectal cancer with at least 12 lymph nodes assessed |
| F_18 The rate of anastomotic leakage in colon and rectal cancer |
| F_19 Assessment of the number of lymph nodes removed during prostatectomy |
| F_20 The percentage of pelvic lymphadenectomy performed according to anatomical ranges |
| F_21 Amounts of positive postoperative margins after prostatectomy |
| F_22 Percentage of patients with suspected lung cancer consulted by a pulmonologist within 14 working days from the date of registering the referral with the service provider |
| F_23 The proportion of patients with mediastinal lymphadenopathy greater than 10 mm who underwent EBUS-TBNA |
| F_24 The proportion of patients with suspected lung cancer and pleural effusion diagnosed with fluid etiology |
| F_25 The proportion of patients with stage III non-small cell lung cancer  who received concurrent chemoradiotherapy |
| F_26 The proportion of ovarian cancer patients treated with primary optimal or suboptimal cytoreduction (no residual mass or ≤1 cm) |
| F_27 The proportion of patients with ovarian cancer who received neoadjuvant chemotherapy |
| F_28 Percentage of patients with ovarian cancer who underwent exploratory laparotomy |
| F_29 The proportion of patients with non-infiltrating breast neoplasms not larger than 2 cm in diameter (excluding patients with BRCA1 and BRCA2 mutations) undergoing breast-conserving therapy |
| F_30 The proportion of patients with infiltrative breast neoplasm not exceeding 3 cm in diameter (total size, including the DCIS component; after excluding patients with BRCA1 and BRCA2 mutations) undergoing breast-conserving treatment |
| F_31 Percentage of diagnostic tests requiring re-description or  re-verification of the material over a 6-week period (computed  tomography, pathomorphological assessment, molecular assessment),  shown for each participating center, by tumor type and test type |
| F_32 Percentage of DCIS breast patients with no axillary lymphadenectomy |
| F_33 The proportion of patients with invasive breast neoplasm without  lymph node metastases (pN0) without axillary lymphadenectomy |
| F_34 Percentage of patients with ER-positive and PR-positive infiltrating breast cancer who received hormone therapy |
| F_35 The percentage of patients with inflammatory or locally advanced, unresectable breast cancer  *** measures analyzed in the presented study** |

Supplemental table S1. List of measures used in KSO`s pilot program
