# Supplementary material for: Analysis of lung cancer measures of the National Cancer Network pilot study in Poland for potential improvement in the quality of advanced-stage lung cancer therapy
Source: BMC Cancer. 2021 Nov 20;21:1252. doi: 10.1186/s12885-021-08994-z (PMC8605770; doi:10.1186/s12885-021-08994-z)
Supplement: Supplementary file 2 — Additional file 2. [file 12885_2021_8994_MOESM2_ESM.pdf]

Dane Pacjenta: Imię: [redacted] Nazwisko: [redacted] Rozpoznanie: C34.8

PESEL [redacted] Miejsce zamieszkania: [redacted]

Wrocław dnia 22-09-2021 r.

OŚWIADCZENIE

Zasady Pilotażu

1. Pilotaż - program którego celem jest ocena organizacji, jakości i efektów opieki onkologicznej w ramach sieci onkologicznej na terenie wybranych województw
2. Pilotażem zostają objęci pacjenci (dalej: Świadczeniobiorcy), u których w okresie realizacji Pilotażu rozpoznano nowotwór złośliwy: gruczołu krokowego, jajnika, jelita grubego, piersi, płuca, wtórny płuc (C78.0) lub przedinwazyjny rak piersi DCIS (D05).
3. W ramach Pilotażu zbierane są informacje o świadczeniach opieki zdrowotnej, profilaktyce, a także satysfakcji pacjentów i ich analiza.
4. Oczekiwanymi efektami pilotażu są poprawa jakości i bezpieczeństwa leczenia onkologicznego oraz wzrost poziomu satysfakcji pacjenta.

Oświadczam, że zapoznałam/em się z powyższymi informacjami i wyrażam zgodę na objęcie pilotażem opieki w ramach sieci onkologicznej, zgodnie z zasadami określonymi w Rozporządzeniu Ministra Zdrowia z dnia 13 grudnia 2018 r. w sprawie programu pilotażowego opieki nad świadczeniobiorcą w ramach sieci onkologicznej (Dz. U. 2018, poz. 2423 z późn. zm.). Niniejsza zgoda ma zastosowanie wyłącznie w przypadku potwierdzenia u mnie nowotworu złośliwego, w tym także, w wyniku przeprowadzonego w oddziale, zabiegu diagnostyczno-leczniczego.

Oświadczam, że zostałem poinformowana/y o zasadach obsługi, przysługujących mi prawach i obowiązkach oraz o zakresie realizowanych świadczeń w ramach programu pilotażowego.

…………………………………..………….

Data i podpis pacjenta

Szczegółowe zasady przetwarzania danych osobowych w pilotażu

Szczegółowe zasady przetwarzania danych osobowych w pilotażu

1. Administratorem danych osobowych jest Dolnośląskie Centrum Onkologii we Wrocławiu, pl. Hirszfelda 12, 53-413 Wrocław.
2. Dane osobowe przetwarzane będą w celu realizacji celów zdrowotnych tj. profilaktyka zdrowotna, udzielanie świadczeń zdrowotnych (diagnostycznych i leczniczych), w tym prowadzenie dokumentacji medycznej, zapewnienia opieki zdrowotnej oraz zarządzania systemami i usługami opieki zdrowotnej, zapewnienia zabezpieczenia społecznego oraz zarządzania systemami i usługami zabezpieczenia społecznego.
3. Podanie danych osobowych w celu realizacji świadczeń medycznych jest obligatoryjne na mocy odrębnych przepisów prawa. Podanie danych dodatkowych (np. nr tel., email) na podstawie udzielonej zgody jest dobrowolne i przysługuje mi możliwość jej modyfikacji albo wycofania w każdym czasie.
4. Pełne Informacje o przetwarzaniu danych osobowych i prawach świadczeniobiorców dostępne są na stronie internetowej http://www.dco.com.pl/ w zakładce Ochrona danych osobowych.

Oświadczam, że zostałem poinformowana/y o warunkach i zakresie przetwarzania danych osobowych, w tym danych medycznych.

Wyrażam zgodę na przetwarzanie tych danych w zakresie niezbędnym do realizacji założeń programu pilotażowego sieci onkologicznej oraz przekazywania tych danych pomiędzy Ośrodkami Współpracującymi, Wojewódzkim Ośrodkiem Koordynującym oraz Narodowym Funduszem Zdrowia.

…………………………………..………….

Data i podpis pacjenta
